# A homozygous *FITM2* mutation causes a deafness-dystonia syndrome with motor regression and signs of ichthyosis and sensory neuropathy

**DOI:** 10.1242/dmm.026476

**Published:** 2017-02-01

**Authors:** Celia Zazo Seco, Anna Castells-Nobau, Seol-hee Joo, Margit Schraders, Jia Nee Foo, Monique van der Voet, S. Sendhil Velan, Bonnie Nijhof, Jaap Oostrik, Erik de Vrieze, Radoslaw Katana, Atika Mansoor, Martijn Huynen, Radek Szklarczyk, Martin Oti, Lisbeth Tranebjærg, Erwin van Wijk, Jolanda M. Scheffer-de Gooyert, Saadat Siddique, Jonathan Baets, Peter de Jonghe, Syed Ali Raza Kazmi, Suresh Anand Sadananthan, Bart P. van de Warrenburg, Chiea Chuen Khor, Martin C. Göpfert, Raheel Qamar, Annette Schenck, Hannie Kremer, Saima Siddiqi

**Affiliations:** 1 Department of Otorhinolaryngology, Hearing and Genes, Radboud University Medical Center, Nijmegen 6525GA, The Netherlands; 2 The Radboud Institute for Molecular Life Sciences, Radboud University Medical Center, Nijmegen 6525GA, The Netherlands; 3 Department of Human Genetics, Radboud University Medical Center, Nijmegen 6525GA, The Netherlands; 4 Donders Institute for Brain, Cognition and Behaviour, Radboud University Medical Center, Nijmegen 6525GA, The Netherlands; 5 Department of Cellular Neurobiology, University of Göttingen, Göttingen 37077, Germany; 6 Human Genetics, Genome Institute of Singapore, Agency for Science, Technology and Research, Singapore 138672, Singapore; 7 Laboratory of Molecular Imaging, Singapore Bioimaging Consortium, A*STAR, Clinical Imaging Research Centre, NUS-A*STAR, Singapore 138667, Singapore; 8 Singapore Institute for Clinical Sciences, A*STAR, Clinical Imaging Research Centre, NUS-A*STAR, Singapore 117609, Singapore; 9 Institute of Biomedical and Genetic Engineering (IBGE), Islamabad 44000, Pakistan; 10 Center for Molecular and Biomolecular Informatics, Radboud University Medical Center, Nijmegen 6525GA, The Netherlands; 11 Department of Molecular Developmental Biology, Radboud University, Nijmegen 6525GA, The Netherlands; 12 Wilhelm Johannsen Centre for Functional Genome Research, Department of Cellular and Molecular Medicine (ICMM), The Panum Institute, University of Copenhagen, Copenhagen 2200, Denmark; 13 Department of Otorhinolaryngology, Head and Neck Surgery and Audiology, Bispebjerg Hospital/Rigshospitalet, Copenhagen 2400, Denmark; 14 Clinical Genetic Clinic, Kennedy Center, Copenhagen University Hospital, Rigshospitalet, Glostrup 2600, Denmark; 15 National Institute of Rehabilitation Medicine (NIRM), Islamabad 44000, Pakistan; 16 Neurogenetics Group, VIB-Department of Molecular Genetics, University of Antwerp, Antwerp 2610, Belgium; 17 Department of Neurology, Antwerp University Hospital, Antwerp 2000, Belgium; 18 Laboratories of Neurogenetics and Neuropathology, Institute Born-Bunge, University of Antwerp, Antwerp 2000, Belgium; 19 Department of Neurology, Radboud University Medical Center, Nijmegen 6525GA, The Netherlands; 20 Singapore Eye Research Institute, Singapore 168751, Singapore; 21 Department of Biochemistry, Yong Loo Lin School of Medicine, National University of Singapore, Singapore 168751, Singapore; 22 COMSATS Institute of Information Technology, Islamabad 45550, Pakistan; 23 Al-Nafees Medical College & Hospital, Isra University, Islamabad 45600, Pakistan

**Keywords:** FITM2, Lipid droplets, *Drosophila*, Hearing impairment, Motor development, Dystonia

## Abstract

A consanguineous family from Pakistan was ascertained to have a novel deafness-dystonia syndrome with motor regression, ichthyosis-like features and signs of sensory neuropathy. By applying a combined strategy of linkage analysis and whole-exome sequencing in the presented family, a homozygous nonsense mutation, c.4G>T (p.Glu2*), in *FITM2* was identified. FITM2 and its paralog FITM1 constitute an evolutionary conserved protein family involved in partitioning of triglycerides into cellular lipid droplets. Despite the role of FITM2 in neutral lipid storage and metabolism, no indications for lipodystrophy were observed in the affected individuals. In order to obtain independent evidence for the involvement of *FITM2* in the human pathology, downregulation of the single *Fitm* ortholog, *CG10671*, in *Drosophila melanogaster* was pursued using RNA interference. Characteristics of the syndrome, including progressive locomotor impairment, hearing loss and disturbed sensory functions, were recapitulated in *Drosophila,* which supports the causative nature of the *FITM2* mutation. Mutation-based genetic counseling can now be provided to the family and insight is obtained into the potential impact of genetic variation in *FITM2*.

## Abstract (precis)

**Editors' choice:** Loss of FITM2 function in humans causes syndromic hearing loss without any signs of a lipodystrophy, although FITM2 is known to function in lipid droplet synthesis and metabolism.

## INTRODUCTION

Hearing involves the transformation of sounds into electrical signals by the inner ear and the subsequent processing of these signals along the central auditory pathways. Mutations in over a hundred genes cause auditory malfunction and hearing impairment (http://hereditaryhearingloss.org/). Defects in the proteins that function in the inner ear can give rise to hearing impairment only (non-syndromic) or, as the function of implicated proteins is often not limited to the auditory system, they can result in multisystem disorders (syndromic hearing impairment).

Deafness–dystonia syndromes are among the more than 400 syndromic forms of hearing impairment described to date (Toriello et al*.*, 2004; Kojovic et al*.*, 2013a). Deafness–dystonia is clinically and etiologically heterogeneous and in many of the investigated cases the underlying causes remain elusive (Kojovic et al*.*, 2013a,b). For some of the cases with a causative mutation identified, disruption of energy homeostasis and/or metabolism are emerging as a common theme. This is true for Mohr–Tranebjaerg syndrome (MIM# 304700, http://www.ncbi.nlm.nih.gov/omim) with mutations in *TIMM8A* (MIM# 300356) (Jin et al*.*, 1996), and for a number of rare mitochondrial disorders with mutations in mitochondrial genes as well as for *SUCLA2*-associated disease (MIM #612073) (Carrozzo et al*.*, 2007).

Cellular energy can be stored as neutral lipids in specialized organelles, the lipid droplets (LDs) (Walther and Farese, 2012). LDs also function in the modulation of cellular signaling, lipid metabolism, transcriptional regulation, autophagy and immunity (Welte, 2015). Defects in genes that affect LD biogenesis and/or function can be associated with hereditary lipodystrophies or motor neuropathies without obvious effects on lipid storage and metabolism (Fujimoto and Parton, 2011). Seipin, for example, which is encoded by *BSCL2* (MIM# 606158), is an endoplasmic reticulum (ER) protein involved in LD formation and maintenance as well as in adipocyte differentiation (Cui et al*.*, 2011;Tian et al*.*, 2011). Loss-of-function mutations in *BSCL2* lead to Berardinelli–Seip congenital lipodystrophy (MIM# 269700), whereas gain-of-toxic-function mutations in *BSCL2* cause a motor neuron disease (MIM# 600794) (Ito and Suzuki, 2009; Magré et al*.*, 2001; Yagi et al*.*, 2011).

The fat storage-inducing transmembrane (FITM) protein family consisting of two conserved proteins, FITM1 and FITM2, is involved in LD partitioning and energy metabolism (Miranda et al*.*, 2011; Kadereit et al*.*, 2008; Gross et al*.*, 2010, 2011; Choudhary et al*.*, 2015). *FITM1* (MIM# 612028) is primarily expressed in skeletal muscle and, at lower levels, in heart. *FITM2* (MIM# 612029) is ubiquitously expressed at low levels in brain, placenta, skeletal muscle, heart, kidney, pancreas, liver, lung, spleen and colon (Kadereit et al*.*, 2008). Expression of FITM proteins in human adipose tissue has not been described yet. In mouse, however, *Fit2* expression is demonstrated to be highest in brown and white adipose tissues (Kadereit et al*.*, 2008). Deficiency of Fit2 in mouse adipose tissue results in progressive lipodystrophy and postnatal whole body *Fit2* knockout is lethal (Miranda et al*.*, 2014; Goh et al*.*, 2015). *FITM2* is part of the *FITM2–R3HDML–HNF4A* locus that is associated with type 2 diabetes, but no phenotypes in humans have hitherto been ascribed specifically to either of the two *FITM* genes (Cho et al*.*, 2012).

In this study, we identified a homozygous truncating mutation in *FITM2* in a consanguineous family of Pakistani origin with Siddiqi syndrome, a novel and characteristic combination of clinical features of progressive sensorineural hearing impairment, delayed development and regression of motor skills, dystonia, low body mass index (BMI), an ichthosis-like appearance of the skin and signs of a sensory neuropathy. No indications of a lipodystrophy were present in the affected individuals. RNAi-induced gene downregulation in *Drosophila melanogaster* recapitulated several aspects of the human phenotype, supporting the link between the syndrome and mutations in *FITM2*.

## RESULTS

### Clinical and paraclinical evaluations of the family

#### Clinical observations of affected individuals

**Fig. 1.**
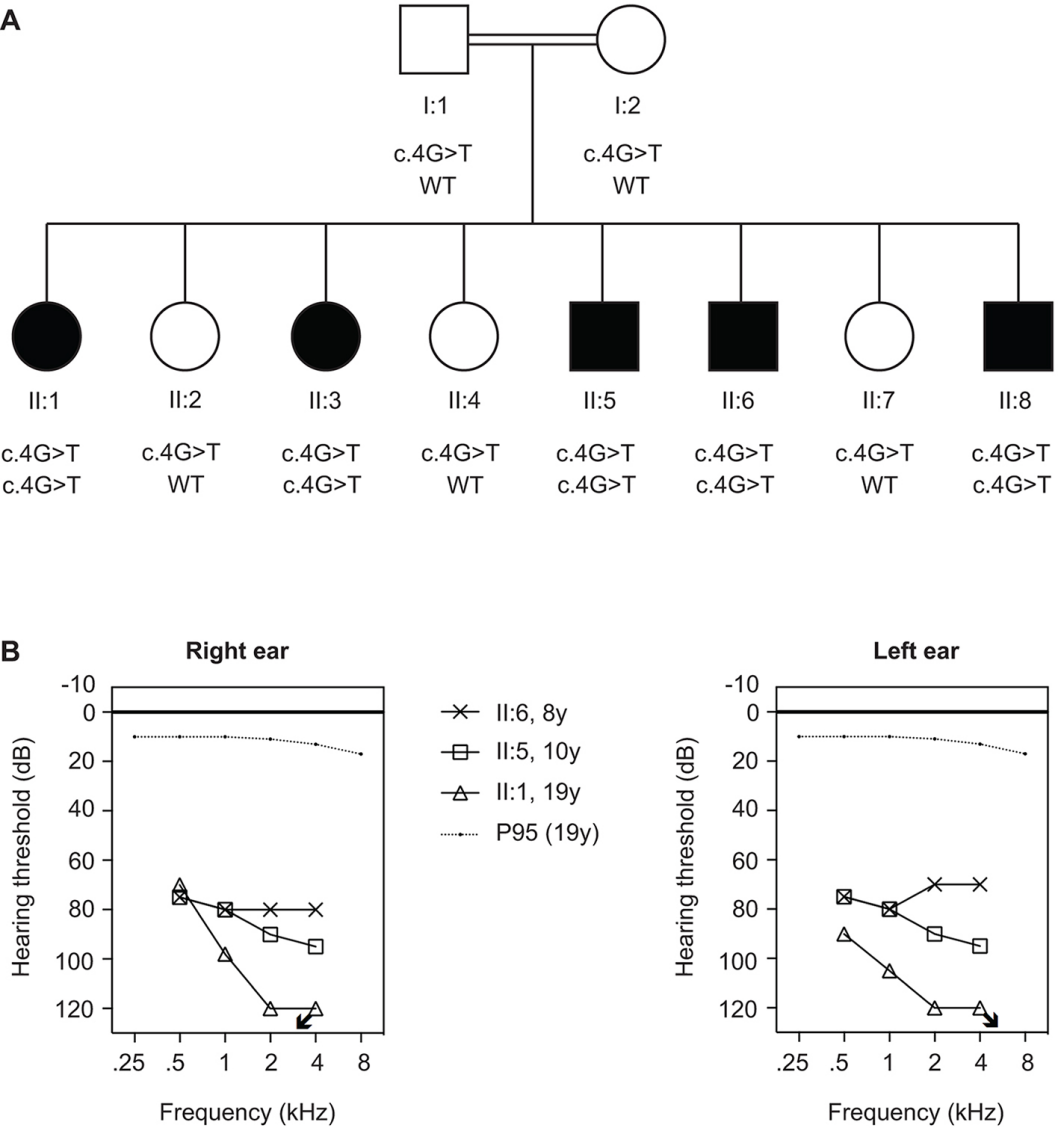
**Pedigree of family W09-1008 and results of pure tone audiometry.** (A) Pedigree of the family identified with Siddiqi syndrome and segregation of the c.4G>T (p.Glu2*) mutation in *FITM2*. (B) Pure tone audiometry of individuals II:1, II:5 and II:6. Age (years) is indicated with the symbol keys. The p95 lines indicate that 95% of individuals of 19 years old have thresholds lower than these. The arrows indicate that the thresholds are lower than 120 dB.

A consanguineous family was identified from the Punjab region in Pakistan with five siblings affected by syndromic hearing impairment and three healthy siblings and parents (Fig. 1A). All affected individuals had global developmental delay and subsequent neuro-regression. Sensorineural hearing impairment was the first symptom of the disease at the age of about six months, and progressed to profound in about ten years (Fig. 1B). No intervention had been undertaken for the hearing impairment of the affected individuals, whose speech was limited to single words. Delayed motor development was evident in all five affected individuals. Four of them only walked independently at the age of three years, whereas individual II:6 never walked independently. The three oldest affected individuals displayed regression in their motor skills from six years of age, with a gradual loss of head control and the ability to sit and walk by the age of ten years. Fine motor skills were poor due to dystonic hand movements and finger deformities. Affected individuals were able to feed themselves but needed assistance in other daily living tasks. Significant dystonic limb movements were present in three cases and truncal dystonia was observed in individuals II:5 and II:8. Contractures, including *pes cavus* deformities, were seen in all three dystonic individuals due to long-standing immobility and dystonia.

There were no signs of spasticity. Muscle wasting of the lower limbs was observed, but given the results of neurophysiological measurements this might be more likely to result from immobility rather than from primary myopathy or motor neuropathy. All affected individuals had sensory complaints; two had non-specific pain in their joints and the remaining three experienced paraesthesia or ‘burning sensation’ in their limb peripheries, joints and trunk. Pain sensation was tested in individuals II:5 and II:6 and found to be absent in the upper limbs and face but preserved in the trunk and lower limbs. Seizures were experienced only by individual II:1 from the age of 15 years.

All five affected individuals displayed ichthyosis-like whitish scaling of the skin with more prominent abnormalities on the shin and scarring alopecia. All five individuals also failed to thrive and had low weights. They did not display dysmorphic features and their daily life behavior did not suggest severe cognitive dysfunction or visual abnormalities. The salient clinical features of the affected individuals are summarized in Table 1.

**Table 1.**
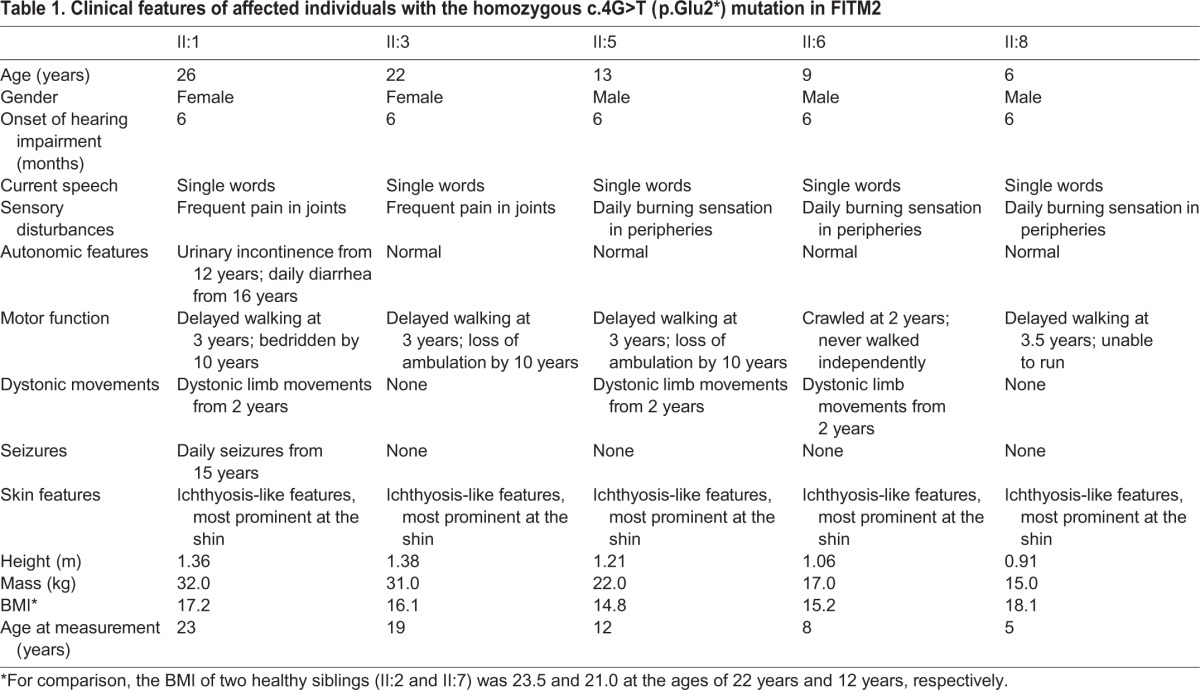
Clinical features of affected individuals with the homozygous c.4G>T (p.Glu2*) mutation in FITM2

#### Clinical examinations of affected individuals

Otological examination, tympanometry and pure-tone audiometry were performed in individuals II:1, II:5 and II:6 at the ages of 19, 10, and eight years, respectively. No external or middle ear abnormalities were noticed and tympanograms were normal for both ears. Pure-tone audiograms displayed bilateral, symmetric, severe or profound hearing impairment, which is sensorineural as bone conduction thresholds were in accordance with air conduction thresholds (Fig. 1B). Brainstem-evoked response audiometry (BERA) was performed for II:1 and II:5 and did not reveal any waveforms up to 90 dB, for both ears.

There were no signs of muscle damage, liver or kidney dysfunction as serum levels of glutamic oxaloacetic transaminase (SGOT), creatine phosphokinase (CPK), lactate dehydrogenase (LDH) and aldolase were found to be within the normal range (Table 2). Fasting glucose levels were determined to be normal as well as fasting serum levels of triglycerides (Table 2).

**Table 2.**
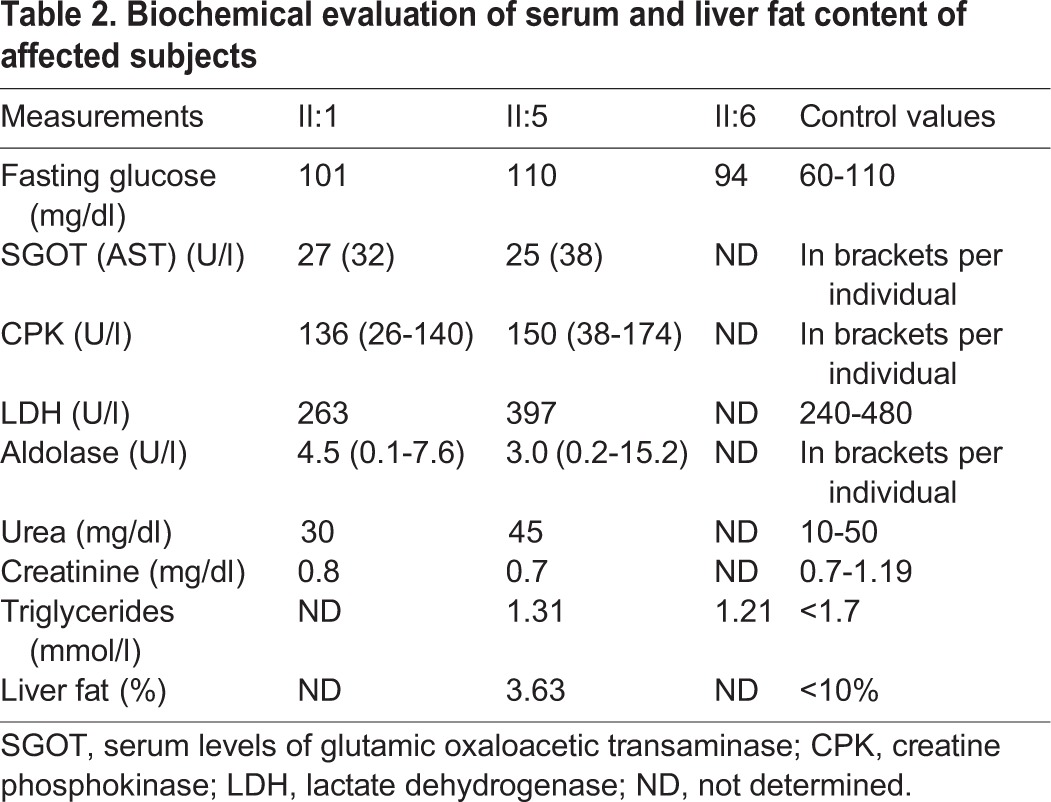
**Biochemical evaluation of serum and liver fat content of affected subjects**

Nerve conduction studies were performed for individual II:1 at the age of 14 years. For the left tibial nerve, small compound muscle action potentials (CMAPs) were measured with normal motor conduction velocity. Normal CMAP and motor conduction velocities were observed in the left median and ulnar nerves. Also, sensory action potentials and conduction velocities in the left median and ulnar nerves were found to be normal. Needle EMG was normal in deltoid, anterior tibial and gastrocnemius muscles. Findings were interpreted as resulting from decreased muscle bulk.

MRI of the abdomen of individuals II:5 and II:6, and of I:1 as a control did not demonstrate any signs of lipodystrophy. Values of liver fat content and subcutaneous adipose tissue (SAT) and visceral adipose tissue (VAT) volumes were in the normal range. MRI of the brain with particular attention to the basal ganglia was performed for individuals II:1 and II:5 and showed no abnormalities, neither in the basal ganglia nor in other regions. A skeletal muscle biopsy (II:1) did not reveal myopathic or neurogenic changes.

In summary, the syndrome in the family is characterized by a novel combination of features: progressive hearing impairment, delayed development and subsequent regression of motor skills, dystonia and low BMI. In addition, ichthyosis-like skin changes are associated with this phenotype and there is a suggestion of small fiber neuropathy (burning sensations, non-length-dependent distribution of sensory abnormalities, normal sensory conduction studies). We propose to call the syndrome ‘Siddiqi syndrome’ after Dr Saima Siddiqi, who initiated the research in this family.

### Whole-exome sequencing identified a nonsense mutation in *FITM2*

Homozygosity mapping and linkage analysis of all family members revealed a single homozygous region of 8.4 Mb on chromosome 20q12–q13.2 (rs2903624–rs6096425) (Table S2). LOD score calculations using 58,023 independent SNPs genome-wide in linkage equilibrium (pairwise r^2^ for each SNP <0.1) revealed a maximum LOD score of 4.00 (Fig. S1). Prolonged ancestral consanguinity that might reduce the significance of linkage peaks is highly unlikely as shown by the percentage of the genomes present in homozygous runs of SNPs (>1 Mb) and the pairwise checks for familial relationships (Table S3, see also Fig. S2).

**Fig. 2.**
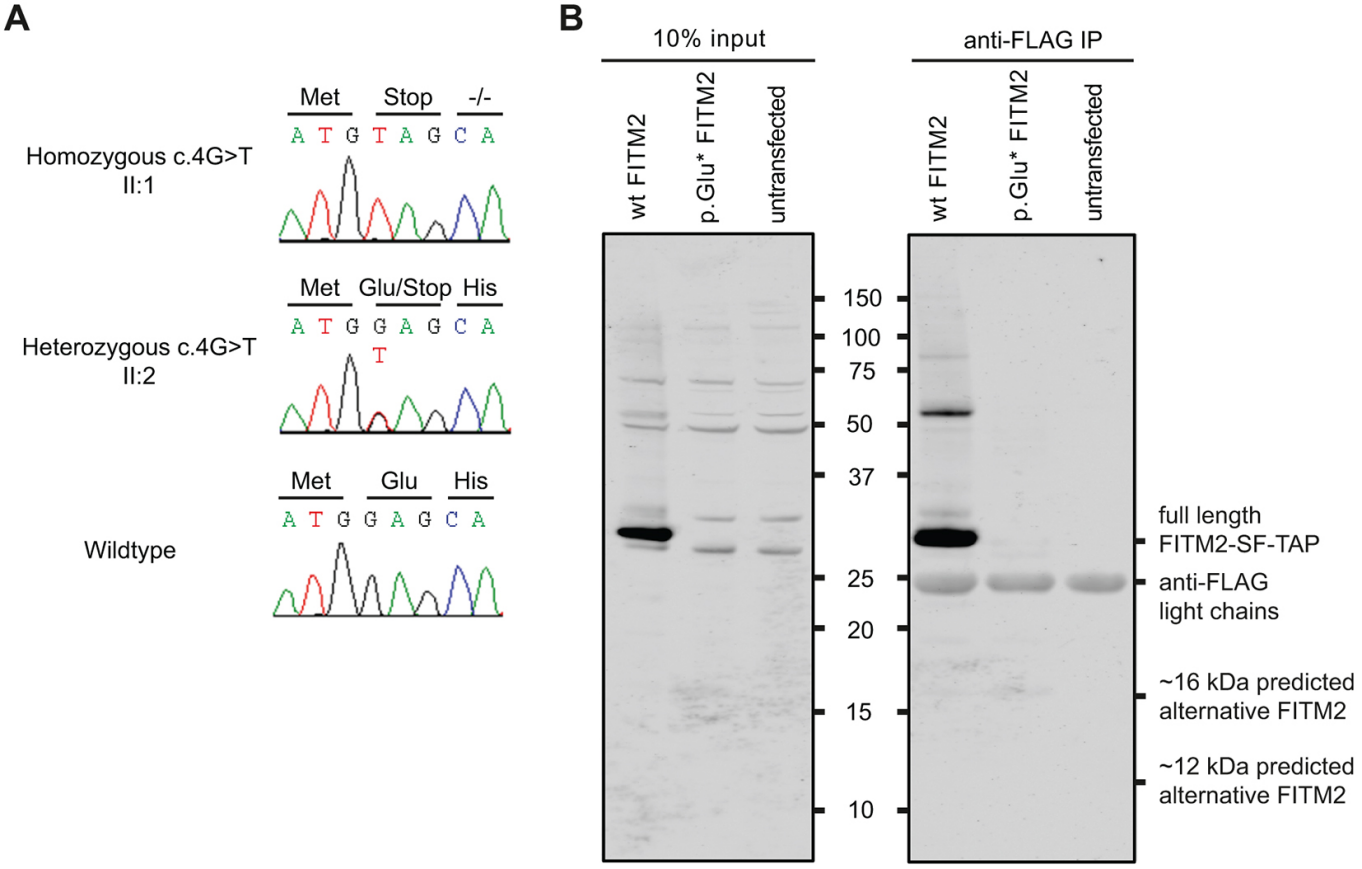
Identification of a genetic defect underlying syndromic hearing impairment in family W09-1008 and expression analysis of wild-type and p.Glu2* FITM2 fused to a FLAG-tag in HEK293T cells. (A) Partial sequences of *FITM2* exon 1 are shown from an affected member, an unaffected heterozygous sib and an unaffected wild-type sib of family W09-1008. The predicted amino acid changes and the surrounding amino acids are indicated above the sequence. Sequence NM_001080472.1 was employed as a reference. (B) The left panel shows a western blot of a gel on which 10% of the cell lysate was loaded (before affinity purification). The right panel shows a western blot of a gel on which 10 µl of the lysate after affinity purification (anti-FLAG) was loaded. Wild-type (WT) FITM2 migrates around 29 kDa and it is absent upon transfection and expression of the p.Glu2* FITM2 construct. After affinity purification, a very weak band is observed at ∼16 kDa. However, the intensity of the 16 kDa band is about 2700-fold lower than the wild-type FITM2 band and therefore it is likely to have little or no biological impact. The gel was immunostained with an anti-FLAG polyclonal antibody. Of four ATG-triplets in the original reading frame, three (codon positions 94, 493 and 508) are predicted to be potential translation initiation sites by the Netstart 1.0 algorithm (Pedersen et al*.*, 1997). Accordingly, alternative proteins would consist of 285 amino acids (aa) (26.2 kDa), 152 aa (11.2 kDa), and 147 aa (10.6 kDa), respectively. Expression constructs encode FITM2 fused to a C-terminal Strep-FLAG-tag (SF-TAP), adding approximately 6 kDa to the proteins. Wild-type FITM2 was found to migrate according to molecular weight of ∼29 kDa, which is lower than the predicted mass of the complete protein. However, fragments of similar length are observed with anti-FITM2 staining in the work of Duckert et al. (2004) and pro-peptide cleavage is predicted by the ProP algorithm. Marker size is indicated between the panels and given in kDa.

The only linkage region contained 125 genes [USCS Genome Browser (https://genome.ucsc.edu/), reference sequence hg19]. Whole-exome sequencing (WES) was performed in the non-affected parents (I:1, I:2) and in two affected siblings, (II:5, II:6). The single homozygous 8.4 Mb region on chromosome 20q12–q13.2 was fully covered in the enrichment kit. In the linkage region, only non-synonymous exonic and canonical splice-site variants were selected that occurred with a frequency of less than 5% in the 1000 genomes (http://www.1000genomes.org/) and HapMap (hapmap.ncbi.nlm.nih.gov) populations, and that were homozygous in both affected siblings and heterozygous in the parents (Tables S4, S5). This revealed a single homozygous nonsense mutation in the second codon, c.4G>T (p.Glu2*, NM_001080472.1; Fig. 2), of *FITM2* that cosegregated in the family with the disease (Fig. 1A; Table S5). This *FITM2* c.4G>T variant was neither present in 274 Pakistani control alleles nor in whole-genome or -exome databases (see Materials and Methods). In order to exclude other potentially causative variants, especially in genomic regions with a LOD score ≥−2, we selected all variants with a MAF (minor allele frequency) <5% that were heterozygous in the parents and compound heterozygous or homozygous in both affected siblings. These analyses did not unveil any variants potentially associated with the syndrome (Tables S5, S6, Fig. S3).

Since postnatal whole-body *Fit2* knockout in mouse is lethal (Goh et al*.*, 2015), we considered the possibility that the present mutation might not lead to complete loss of FITM2 function, potentially as a result of downstream alternative translation initiation sites. We expressed C-terminally Strep/FLAG-tagged wild-type and p.Glu2* FITM2 in HEK293T cells to identify potential N-terminally truncated FITM2 proteins. Even after FLAG affinity purification, no indications for significant amounts of alternative *FITM2* products were obtained (Fig. 2B). We conclude that with high likelihood the *FITM2* mutation results in a complete loss of *FITM2* function.

To further address the involvement of *FITM2* mutations in hearing impairment syndromes with characteristics overlapping those in the present family, *FITM2* was sequenced in six index individuals with deafness and a sensory polyneuropathy. Also, four index cases were tested who were suspected to be presenting with Mohr–Tranebjaerg syndrome and who did not carry *TIMM8A* mutations. Mutation analysis did not reveal biallelic variants of *FITM2* with allele frequencies <5% in the HapMap, 1000 genomes or Exome Aggregation Consortium database (ExAC; http://exac.broadinstitute.org/) databases.

### *Drosophila* models of *FITM2*

To gain independent support for the role of *FITM2* in the phenotype of the presented family and to dissect the underlying tissue-specific pathologies, we studied FITM function in *Drosophila melanogaster*. The *Drosophila* genome harbors a single, thus far uncharacterized, gene representing the human FITM protein family (FITM1 and FITM2), *CG10671*, which we accordingly name *Fitm*. FITM1 and FITM2 share 25% amino sequence identity with their annotated ortholog *Fitm* (see http://www.ensembl.org/). According to ModEncode and FlyAtlas expression databases (Chintapalli et al*.*, 2007; Graveley et al*.*, 2011), *Fitm* is expressed widely throughout developmental stages and tissues, with highest expression in adult fat body, heart and carcass. The lack of genetic redundancy of *Fitm* in *Drosophila* suggests that its complete absence in a null mutant is likely to lead to lethality in early stages of development. Therefore, we decreased the expression of *Fitm* by constitutive RNA-mediated interference (RNAi) with the *UAS*-GAL4 system. The efficacy of *Fitm* downregulation was determined by qRT-PCR upon ubiquitous knockdown using the *αTub84B*-GAL4 driver. All RNAi lines presented comparable levels of *Fitm* downregulation with respect to the corresponding control line. The decrease in *Fitm* transcripts was 92% (*P*=0.003) for *Fitm* RNAi-1A, 80% (*P*=0.008) for *Fitm* RNAi-1B and 80% for *Fitm* RNAi-2 (*P*=0.008) as compared with the respective control lines (Fig. S4). Since RNAi-1A and -1B carry the same RNAi construct, we prioritized RNAi-1A and RNAi-2 lines for our experiments. For the experiments that did not give conclusive data for one of the tested conditions and allowed the use of females, we additionally investigated the X chromosome-linked RNAi line RNAi-1B.

#### Knockdown of *Drosophila Fitm* causes locomotor impairment

To address locomotor function, impaired in the presented family, we first subjected *Fitm* knockdown models to an explorative negative geotaxis test in which climbing capacity was visually evaluated. Flies with *Fitm* knockdown mediated by the *αTub84B*-GAL4 and *Mef2*-GAL4 drivers displayed a decreased climbing capability at 4, 12 and 21 days after eclosion (Movie 1). The latter driver is highly expressed in muscle cells. Upon *Fitm* knockdown mediated by the fat-body-specific *C7*-GAL4 driver, flies were severely impaired in climbing at day 21, although normal at days 4 and 12 after eclosion. This locomotion phenotype was highly consistent in both RNAi lines tested with ubiquitous, preferential skeletal muscle and fat body promoters. Pan-neuronal knockdown of *Fitm* with the *w*; *UAS*-*Dcr-2*; *elav*-GAL4 driver and *w*, *UAS-Dcr-2*; *n-syb*-GAL4 did not lead to obvious anomalies, flies showed normal climbing behavior.

**Fig. 3.**
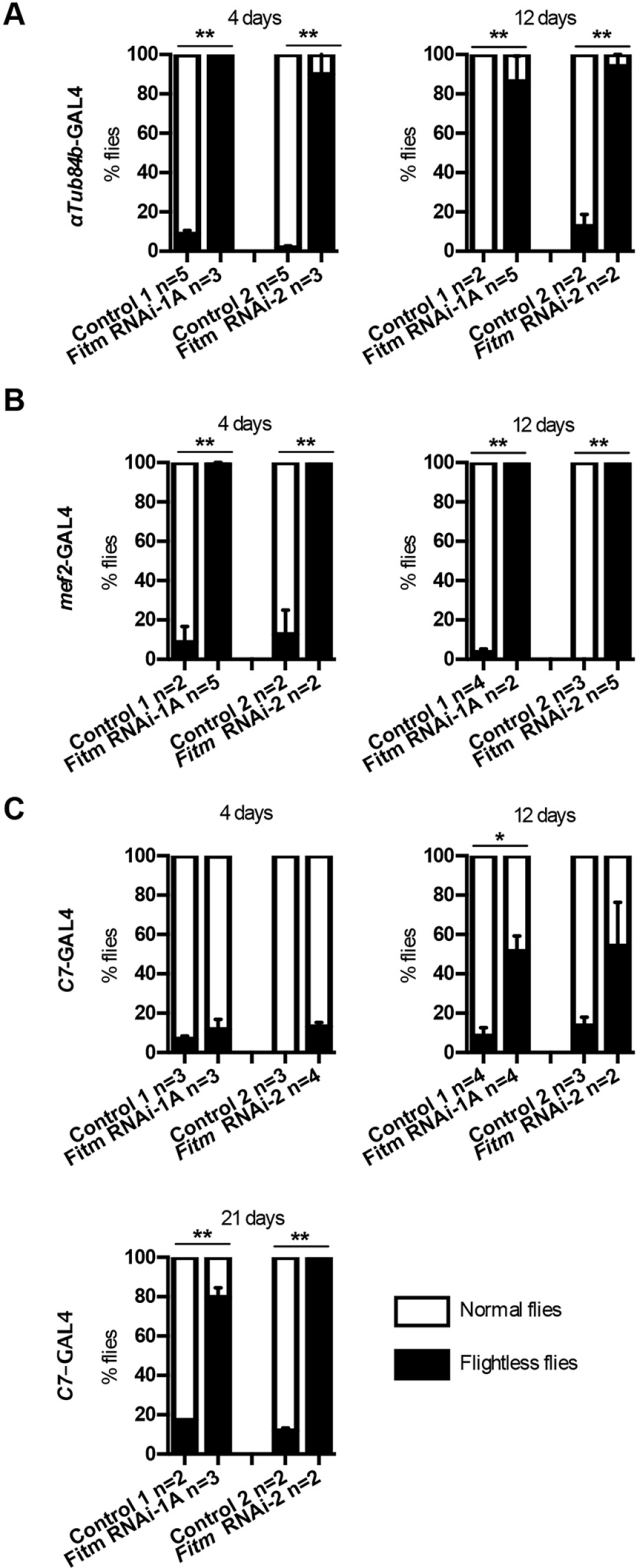
**Knockdown of *Fitm* impairs locomotor abilities in *Drosophila.*** Stacked bar graphs show the average percentage of flightless flies (black bars) and flies with normal flight responses (white bars). Error bars represent s.e.m. The indicated days represent days of age past eclosion. *Fitm* knocked down ubiquitously and preferentially in skeletal muscle with the *αTub84B*-GAL4 (A) and *Mef2*-GAL4 (B) promoters, respectively, and *Fitm* RNAi-1A and *Fitm* RNAi-2 showed significant locomotor impairment at all time points. (C) *Fitm* knockdown in the fat body (*C7*-GAL4 driver) using *Fitm* RNAi-1A and *Fitm* RNAi-2 revealed a progressive locomotor impairment evident at 12 days after eclosion as compared with corresponding age-matched control flies. The percentages of normal and flightless flies per experiment was used to determine statistical differences by one-way ANOVA with Tukey's correction for multiple testing, *P<0.05, **P<0.01. Average percentages are plotted in the graphs. The *n* refers to the number of experiments. Number and percentages of flightless and normal flies in each independent experiment can be found in Table S7.

To further characterize the locomotor abilities of *Fitm* knockdown flies in a quantitative manner, the island assay (Schmidt et al., 2012) was performed using the two driver lines that resulted in impaired climbing in the negative geotaxis test. This revealed that ubiquitous *Fitm* knockdown leads to severe locomotor impairment, resulting in significantly higher numbers of flightless flies at days 4 and 12 after eclosion (*P*<0.0001; Fig. 3A). Upon downregulation of *Fitm* using the *Mef2-*GAL4 driver, more than 98% of the flies displayed a flightless phenotype at 4 and 12 days old (*P*<0.0001 for all analyzed conditions; Fig. 3B). The effect of fat-body-specific *Fitm* knockdown on locomotion was evaluated because of the evolutionary conserved role of *FITM2* in LD biogenesis in adipose tissue (Kadereit et al., 2008; Miranda et al., 2014) and because of high *Fitm* expression in *Drosophila* fat bodies. In agreement with this, a progressive locomotor impairment was observed. A maximum of 13% flightless flies were observed at 4 days past eclosion and a minimum of 51% flightless flies were observed at 12 days, which further raised to more than 78% at 21 days past eclosion (*P*<0.001 for all analyzed conditions; Fig. 3C). Pan-neuronal knockdown of *Fitm* with the *w; Dcr-2*; *elav*-GAL4 and *w*, *UAS-Dcr-2*; *n-syb*-GAL4 drivers did not lead to any significant locomotor impairment in the island assay (Fig. S5).

We visually evaluated body and wing movements of the flightless flies, as sensory motor coordination is essential for flight initiation and a sensory neuropathy is part of the phenotype in the Pakistani family. At the initiation of flight, flies first raise their wings to a stable position that will be held for a few seconds before take-off (Card and Dickinson, 2008). In a subset of *Fitm* knockdown flies, but not in controls, these flight initiation movements were uncoordinated. The knockdown flies failed to upstroke their wings for takeoff and instead displayed fast wing movements, and uncontrolled jumping and shaking of their corpus (Movie 2). Upon pan-neuronal *Fitm* knockdown, flies did not display this phenotype.

In conclusion, loss of *Fitm* expression in *Drosophila* causes locomotor defects and *Fitm* knockdown preferentially in muscle or specifically in the fat body suffices to induce this phenotype.

#### Downregulation of *Fitm* causes abnormal dendrite branching and field coverage of *Drosophila* multi-dendritic sensory neurons

**Fig. 4.**
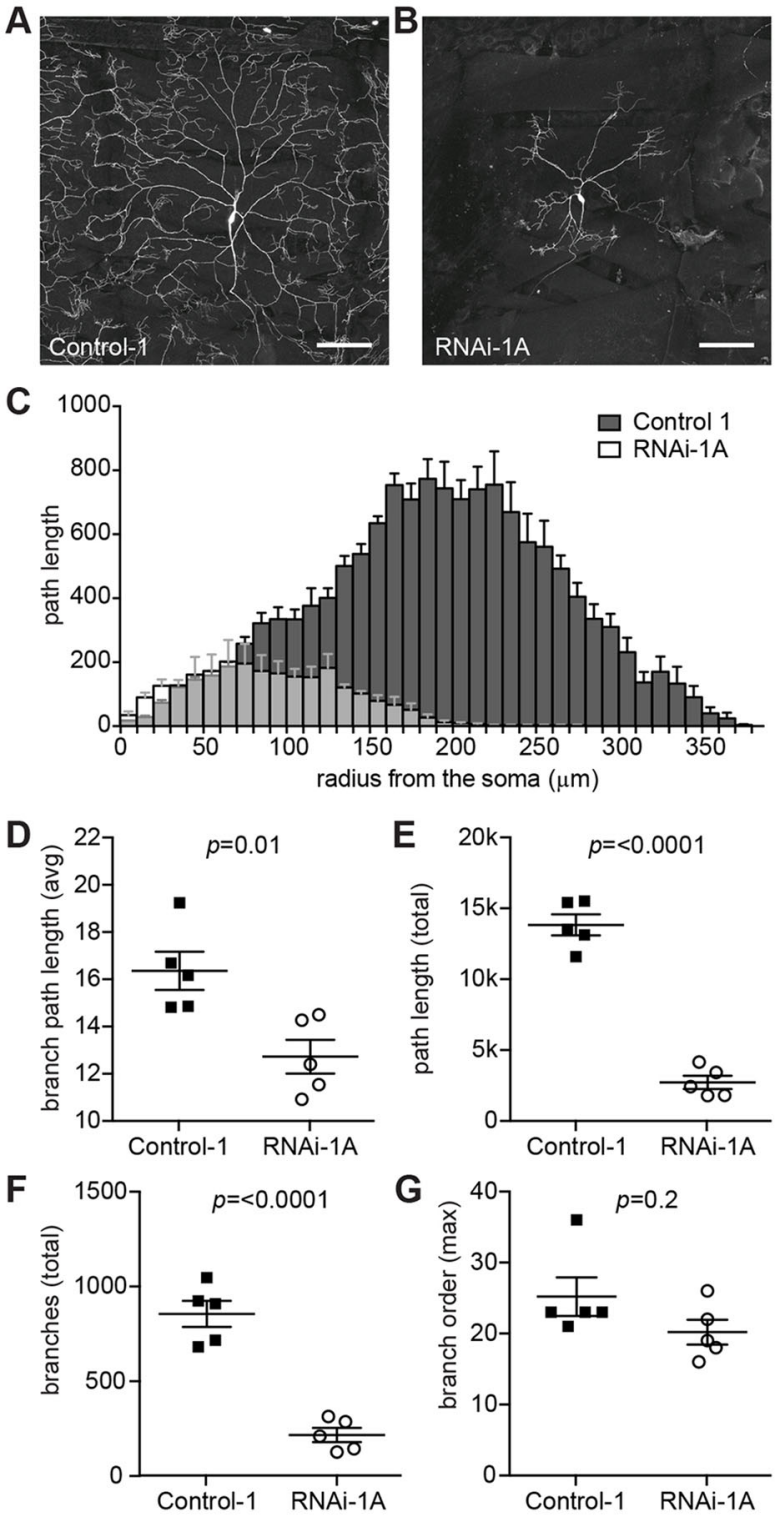
***Fitm* RNAi-1 knockdown in *Drosophila* interferes with morphology of nociceptive multi-dendritic sensory neurons.** (A,B) Confocal projections of class IV da neurons within segment A3 of third instar larvae, visualized with the class IV da-specific drivers *477*-GAL4 and *ppk*-GAL4 and UAS-mCD8::GFP. The ddaC neurons show abnormal dendritic morphology in a subset of *Fitm* RNAi larvae. (A) Control-1 shows ddaC contact to neighboring neurons. (B) Knockdown of *Fitm* in line RNAi-1A results in a severe outgrowth defect, observed in 5/18 larvae. The five highly abnormal neurons (see Fig. S6) were analyzed further, to test whether they significantly differ from the control. (C) Sholl analysis (Wearne et al., 2005) of control (*n*=5) and selected highly abnormal neurons (*n*=5) reveals defects as a measure of the soma distance. The *Fitm* RNAi-1A dendritic field coverage has a radius that is 60% the size of that of the control. (D-G) Quantitative analysis of dendritic trees reveals that the affected *Fitm* RNAi-1 neurons have (D) a reduced average branch path length (*P*=0.01), (E) a reduced accumulative branch path length (*P*≤0.0001), (F) a decreased number of branches (*P*≤0.0001), and (G) not significantly changed maximal branch order (*P*=0.2). Dorsal is up in A,B. Scale bar: 100 μm. Error bars in (D-G) indicate s.e.m. *t*-test between control and knockdown conditions was performed for each parameter to determine significance. *P*-values are depicted in each graph. Five neurons per strain, derived from five different larvae, were analyzed. The data were collected in two independent experiments. For underlying numerical data see Tables S8 and S9. More information about the depicted parameters can be found in Table S11.

As signs of a sensory neuropathy are part of the syndrome caused by a nonsense mutation in *FITM2*, we evaluated the role of *Fitm* in sensory neuron development by inspecting the dorsal class IV dendritic arborization C (ddaC) neurons in third instar larvae. These nociceptive neurons show a complex, but rather stereotypic dendritic branching with a large field of coverage (Fig. 4A) that, together with other class IV dendritic arborization neurons, tile the larval body wall. *Fitm* expression was downregulated by RNAi in class IV dendritic arborization neurons using a combination of the *477-*GAL4 and *ppk-*GAL4 drivers, which simultaneously induce expression of the fluorescent marker *UAS-*mCD8::GFP. A driver line with a combination of two GAL4 elements was used to increase the number of GAL4 molecules to bind *UAS-*mCD8::GFP, *UAS-Fitm* RNAi and *UAS-Dcr-2* to enhance their expression. Knockdown of *Fitm* upon induction of *Fitm* RNAi-1A resulted in a strong reduction of the dendritic field coverage in a subset of larvae (5 of 18 analyzed), with contact with the neighboring sensory neurons being completely absent (Fig. 4B). Fig. S6 shows the obtained microscopic images of the traced neurons and representative images of untraced neurons of knockdown flies that were evaluated as normal. A dendritic field coverage defect phenotype was also observed in a *Fitm* RNAi-2 larva, but only occurred in one of 40 larvae analyzed (Fig. S7A-F). It was, however, never observed in any control larva, either during this or other studies (Mukhopadhyay et al*.*, 2010; Kramer et al*.*, 2011; Klein et al*.*, 2015). Reduced penetrance of RNAi-induced phenotypes is a known phenomenon and could be dependent on the timing and efficiency of knockdown (Mauss et al*.*, 2009; Godenschwege et al*.*, 2006). In our experiment, we used genetic tools and conditions to maximize RNAi efficiency (two driver elements, *UAS-Dcr-2*, and a temperature of 28°C). Alternatively, reduced penetrance can also be observed in null mutants when the function of the affected gene can partially be compensated by others (Raj et al., 2010; Chalancon et al*.*, 2012; Cooper et al*.*, 2013).

To gain more insight in the underlying defects of the abnormal field coverage, we performed manual tracing and quantitative analysis on the control and abnormal *Fitm* RNAi-1A dendritic trees (Fig. 4C-G; Tables S8, S9). Sholl analysis showed that the dendritic field coverage of controls has a maximum radius of 350±21 μm (mean±s.e.m.), the dendritic field coverage in *Fitm* RNAi-1A is 60% the size with a significantly smaller radius of 210±39 μm (*P*=0.0001; Fig. 4C). Analysis of the dendritic trees revealed a reduced average branch path length (*P*=0.01; Fig. 4D), defined as the distance between two branching points, a reduced accumulative branch path length (*P*≤0.0001; Fig. 4E), defined as sum of the distance of all branches contained in a neuron, and a decreased number of branches (*P*≤0.0001; Fig. 4F); all in concordance with the reduced field of coverage. The maximal branch order was not significantly decreased (*P*=0.2; Fig. 4G), defined as the order of a branch with respect to the soma; each branching point will lead to branches with a higher branch order. Although only one *Fitm* RNAi-2 larva was found to be affected, some aspects are similar to the RNAi-1A phenotype. Analysis of the affected *Fitm* RNAi-2 dendritic tree revealed low average branch path length and accumulative branch path length (Fig. S7C,D, respectively), but the number of branches and branch order were high (Fig. S7E,F; Table S10).

Taken together, our results suggest that *Fitm* is required for normal branching and dendritic field coverage in a subset of *Drosophila* ddaC nociceptive sensory neurons.

#### *Fitm* is required for normal hearing in *Drosophila*

**Fig. 5.**
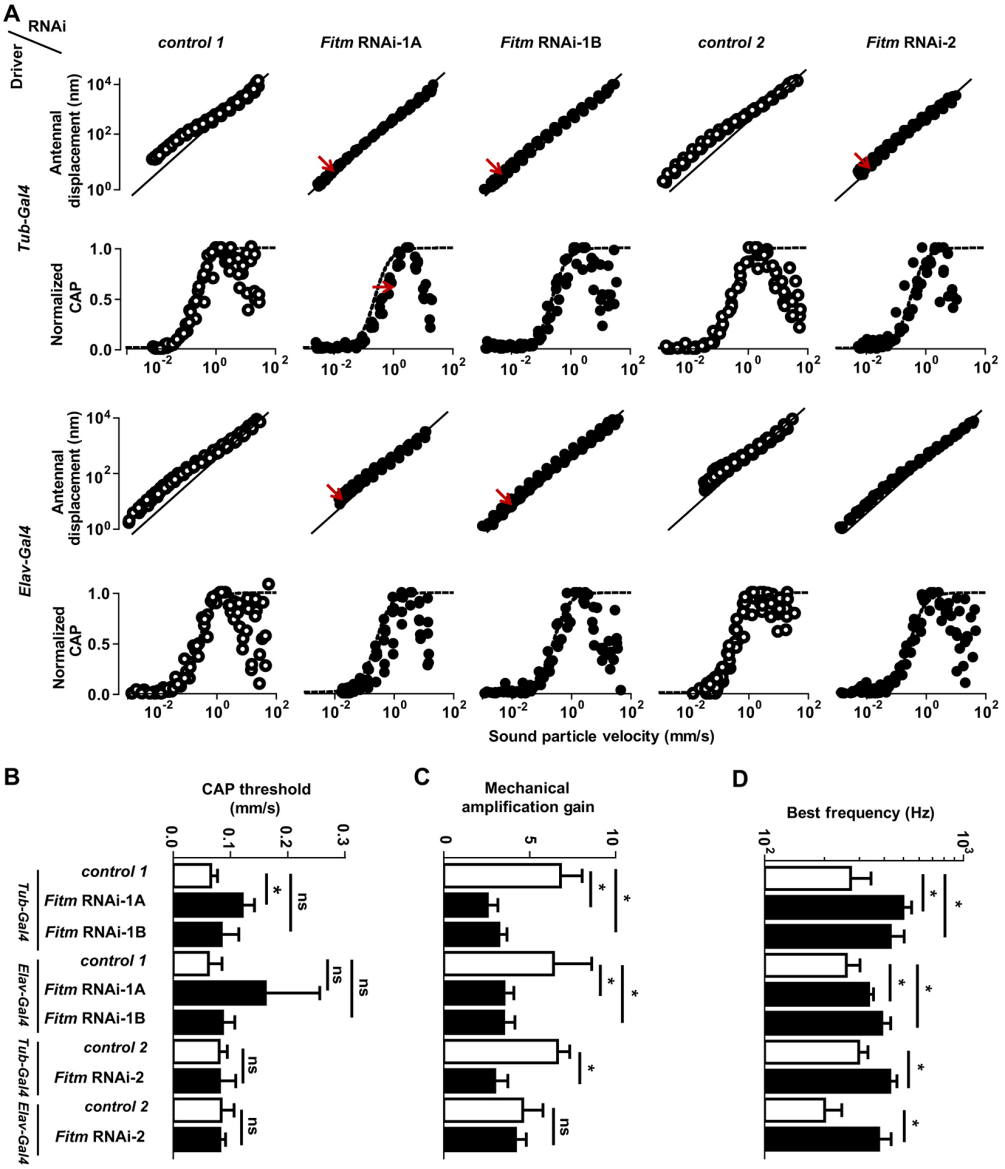
**Knockdown of *Fitm* impairs *Drosophila* hearing.** Antennal vibrations and ensuing antennal nerve potentials were measured in the *Fitm* RNAi-1A, *Fitm* RNAi-1B and *Fitm* RNAi-2 lines and the corresponding controls (Control-1 and Control-2) crossed to the pan-neuronal *elav*-GAL4 and ubiquitous *αTub84B*-GAL4 drivers three days after eclosion. (A) Sound-evoked antennal displacement amplitudes (upper panels, log scale) and normalized compound action potential (CAP) amplitudes as functions of the sound particle velocity. Each circle indicates a single data point. Solid (upper panels) and dashed (lower panels) lines indicate linear auditory mechanics, as observed upon the loss of mechanical amplification by auditory sensory neuron motility (Senthilan et al*.*, 2012), and Hill fits to the pooled CAP responses of each strain, respectively. Red arrows indicate significant differences to controls. (B) Respective CAP thresholds, deduced from Hill fits to the CAP amplitudes of each individual. (C) Respective mechanical amplification gains provided by auditory sensory neuron motility. (D) Respective mechanical best frequencies of the antennal sound receivers, deduced from the mechanical fluctuations in the absence of sound stimulation (Senthilan et al., 2012). Per strain, five flies were analyzed and three independent measures were taken. Each data point represents the average response to 10 stimulus presentations. Error bars indicate s.d. *P<0.05; ns, not significant by two-tailed Mann–Whitney U-tests. If applicable, Bonferroni correction was used to correct for multiple testing. For original values, see Table S12.

Affected members of the presented family displayed postnatal sensorineural hearing impairment that progressed to profound. Therefore, we tested whether *Fitm* is implicated in *Drosophila* hearing by analyzing sound-evoked mechanical and electrical responses of the antennal hearing organ (Fig. 5) upon ubiquitous or pan-neuronal knockdown. To evoke sound responses, we exposed the flies to pure tones of different intensities at the individual mechanical best frequency of their antennal sound receiver (Göpfert et al*.*, 2006). The resulting vibrations of this receiver were measured as well as the ensuing compound action potentials (CAPs) propagated by the axonal projections of the fly's auditory sensory neurons in the antennal nerve (Fig. 5A). In genetic background controls, sound particle velocities exceeding ∼0.05 mm s^−1^ evoked CAP responses (Fig. 5B), consistent with published data on wild-type flies (Senthilan et al*.*, 2012). As in wild-type flies, the sound-induced displacement of the antenna also scaled nonlinearly with the intensity of sound stimulation (Fig. 5A), displaying a compressive nonlinearity that, arising from motile responses of auditory sensory neurons, actively amplified the antennal displacement response to faint sounds with an amplification gain of approximately seven (Fig. 5C). Ubiquitous knockdown of *Fitm* with *Fitm* RNAi-1A significantly increased the threshold of the sound-evoked CAP responses (Fig. 5B; Table S12), documenting a loss in auditory sensitivity. Auditory sensitivity seemed uncompromised by pan-neural knockdown with *Fitm* RNAi-1A and knockdown with *Fitm* RNAi-1B or RNAi-2 (Fig. 5C), yet significant hearing impairment was detected in all three RNAi lines upon ubiquitous knockdown when we examined the nonlinear scaling of their antennal vibrations. RNAi-1A- and RNAi-1B-induced knockdown reduced this nonlinear scaling, significantly lowering the mechanical amplification gain (Fig. 5C; Table S12). Moreover, all three knockout constructs significantly increased the best frequency of the antennal sound receiver (Fig. 5D; Table S12), documenting defects in the active frequency tuning of the receiver, which is achieved through mechanical amplification.

Together, these results document that *Drosophila* auditory sensory neurons require *Fitm* for normal mechanical amplification in hearing, which is linked to auditory stimulus transduction and auditory neuron integrity (Senthilan et al*.*, 2012).

#### *Fitm* is important for lipid droplet size in the fat body of adult *Drosophila*

**Fig. 6.**
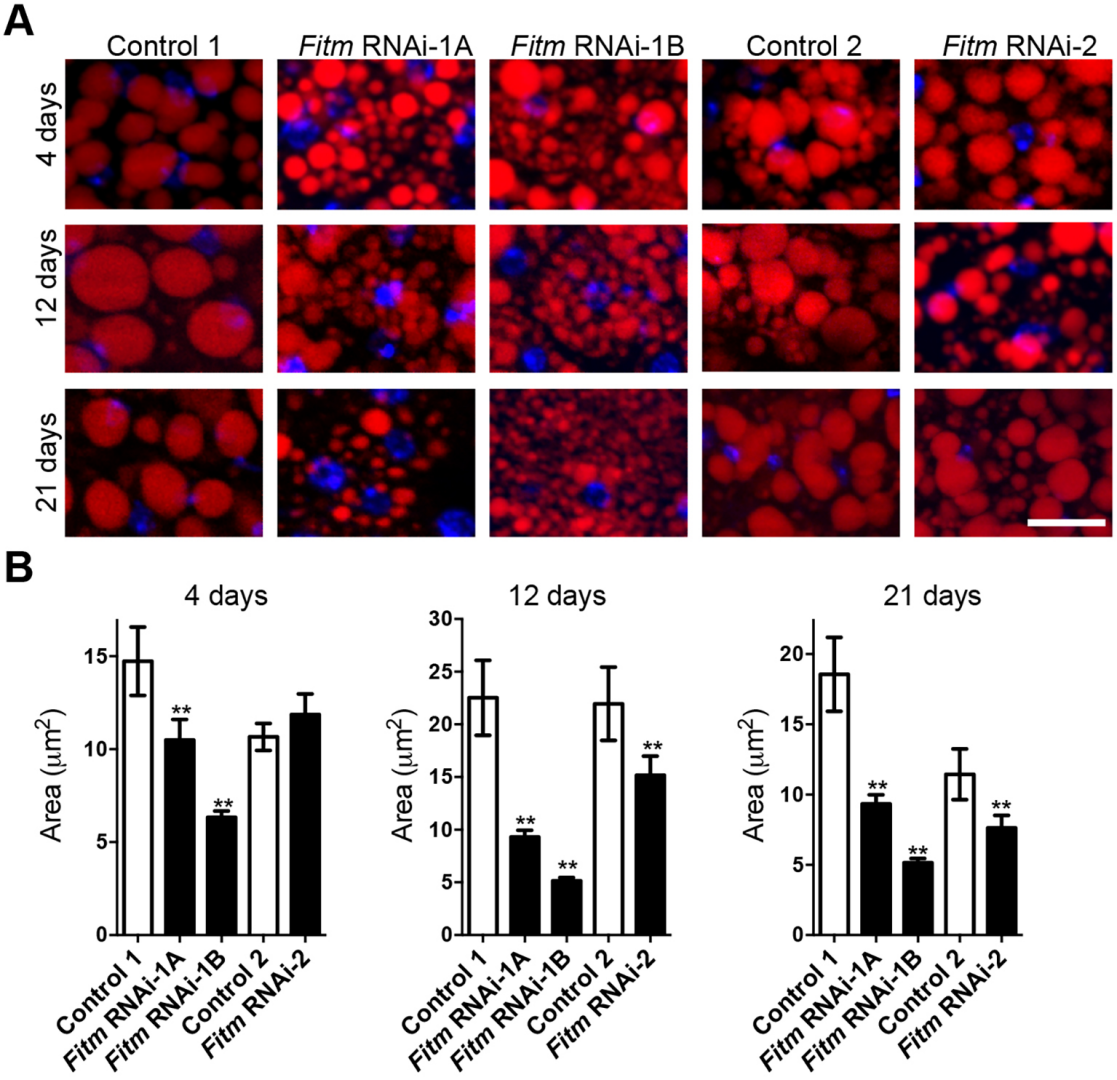
***Fitm* knockdown leads to progressive decrease of lipid droplet size.** (A) Representative images of lipid droplets (LDs) labeled with Bodipy (red) in fat bodies of C7-GAL4-induced *Fitm* knockdown flies (*Fitm* RNAi-1A and *Fitm* RNAi-1B) as compared with the corresponding genetic background control flies at the indicated time points past eclosion. Nuclei were stained with DAPI (blue). Scale bar: 10 µm. (B) LD area is significantly reduced at 4, 12 and 21 days after eclosion upon *Fitm* knockdown with RNAi-1A and RNAi-1B constructs, and with RNAi-2 at 12 and 21 days after eclosion. The graphs display the mean LD area per experimental condition. Error bars represent the 95% confidence interval. **P<0.01 by ANOVA with Tukey correction. The number of images analyzed, the number of female flies per strain selected for this analysis and the mean size of lipid droplet per condition are, respectively, as follows. At 4 days: RNAi-1A (11, 5, 10.49 μm); RNAi-1B (14, 6, 6.33 μm); Control-1 (10, 4, 14.73 μm); RNAi-2 (17, 4, 11.86 μm); Control-2 (22, 6, 10.66 μm). At 12 days: RNAi-1A (38, 5, 9.31 μm); RNAi-1B (33, 6, 5.14 μm); Control-1 (24, 6, 22.52 μm); RNAi-2 (29, 5 15.16 μm); Control-2 (18, 6, 21.95 μm). At 21 days: RNAi-1A (16, 5, 5.14 μm); RNAi-1B (21, 7, 11.49 μm); Control-1 (31, 4, 18.56 μm); RNAi-2B (18, 6, 7.63 μm); Control-2 (32, 6, 11.44 μm). Quantification of lipid droplet size was performed in one experiment. The observation of progressively reduced lipid droplet sizes in the RNAi-lines was made in three independent experiments.

Having shown a number of parallels between human and *Drosophila* phenotypes, we finally sought to evaluate whether *Drosophila Fitm* functions in LD formation, as previously reported in other organisms (Kadereit et al*.*, 2008; Gross et al*.*, 2011; Choudhary et al*.*, 2015; Miranda et al*.*, 2014). We thus knocked down *Fitm* expression in the fat body and evaluated LD size. *Fitm* RNAi-1A and RNAi-1B knockdown flies demonstrated a diminished LD size as compared with flies of the background line at 4, 12 and 21 days after eclosion (Fig. 6). The *Fitm* RNAi-2 knockdown flies exhibited a reduction in LD size that started at 12 days after eclosion (Fig. 6A). Strikingly, all RNAi lines showed a progressive phenotype; the reduction in the LD size was milder or nonexistent in young flies (4 days after eclosion) and more severe in ageing flies (12 and 21 days after eclosion) (Fig. 6A,B).

We conclude that *Fitm* function in LD formation is conserved in *Drosophila* (Kadereit et al*.*, 2008).

## DISCUSSION

We have described a family with a novel homozygous truncating mutation, c.4G>T (p.Glu2*), in *FITM2*. Affected individuals display Siddiqi syndrome, a novel syndrome characterized by progressive sensorineural hearing impairment, delayed motor development and subsequent regression, low BMI, ichthyosis-like skin alterations and signs of a small fiber neuropathy. Dystonia was observed in some of the affected individuals and seizures and chronic diarrhea only in the oldest affected sibling. The chronic diarrhea might well be a symptom of malabsorptive enteropathy which is seen in mouse upon postnatal *Fit2* deletion (Goh et al*.*, 2015). The combination of the disease characteristics is novel, although observed phenotypic characteristics in the family overlap with several known monogenic neurological conditions such as Troyer syndrome (MIM #275900) and deafness-dystonia syndromes including Mohr–Tranebjaerg syndrome (MIM #304700) and Megdel syndrome (MIM #614739). To delineate Siddiqi syndrome, further families with *FITM2* mutations need to be identified and evaluated clinically. Currently, it cannot be excluded that part of the phenotype results from mutations in other genes, especially the characteristics seen in only some of the cases. However, no homozygous rare variants were identified in individuals II:5 and II:6 in autozygous regions (>1 Mb) shared by II:1, II:5 and II:6 only. Also, these regions do not harbor genes known to be associated with dystonia. Similarly, regions uniquely autozygous in II:1 do not harbor potentially pathogenic heterozygous variants in both parents to explain seizures and diarrhea. Defects in known deafness genes that could explain the hearing loss only were also not identified. The causative association of the syndrome with a loss-of-function mutation in *FITM2* is supported by modeling of the disease in *Drosophila melanogaster*, which has been proven to be a suitable model for studying conserved aspects of lipid metabolism and LD biology (Tian et al*.*, 2011; Baker and Thummel, 2007). RNAi knockdown of the single *Drosophila Fitm* ortholog recapitulated hearing impairment, locomotor defects and abnormalities of the sensory system.

Sensorineural hearing impairment is the first symptom of Siddiqi syndrome. The audiometric evaluations did not allow us to discriminate whether the hearing impairment has a cochlear or retrocochlear neuronal origin. The hearing phenotype resulting from *Fitm* knockdown in *Drosophila* reflected impaired auditory stimulus transduction and auditory sensory neuron function, which support a sensorineuronal hearing impairment in the human phenotype. A cochlear component might well contribute to progressive dysfunction of the auditory system in the affected individuals. LDs are prominent constituents of Hensen cells, which are highly specialized cells in the organ of Corti, and these Hensen cell LDs have been suggested to play a role in anti-inflammatory responses to prevent cochlear damage (Merchan et al*.*, 1980; Bell and Fletcher, 2004; Kalinec et al*.*, 2009; Urrutia and Kalinec, 2015). Additionally, a mechanical function in modulating sound detection has been proposed for LDs in Hensen cells (Merchan et al*.*, 1980). However, whether vestibular dysfunction is part of the inner ear phenotype could not be evaluated, and it therefore remains undetermined whether impaired balance contributed to the delayed motor development of the subjects.

Of note is that affected individuals do not have signs of a lipodystrophy, which is in contrast to findings in mice where post-differentiation adipose-specific knockout of *Fit2* results in progressive reduction of white adipose tissue (Miranda et al*.*, 2014). Functional redundancy in human adipose tissue might exist for FITM2 through FITM1, which is apparently not the case in the mouse. In the mouse, FIT2 is prominently expressed in adipose tissue in which FIT1 was not detected (Kadereit et al*.*, 2008). The relative expression levels of *FITM1* and *FITM2* in human adipose tissue is hitherto unknown. A further explanation for the discrepancy in lethality of *FITM2*/*Fit2* loss of function could be the presence of alternative sites of transcription start in humans, resulting in mRNAs that are not affected by the *FITM2* variant. Our experimental setup for detection of alternative translation initiation sites cannot exclude such alternative transcription start sites.

The molecular mechanism(s) underlying Siddiqi syndrome are still elusive but might well be related to one of newly discovered functions of LDs (Welte, 2015; Barbosa et al*.*, 2015). Disturbance of energy metabolism and homeostasis might be part of the underlying mechanism(s), as has been suggested for some other deafness-dystonia syndromes (Jin et al*.*, 1996; Elpeleg et al*.*, 2005; Engl et al*.*, 2012). In this respect, it is interesting that overexpression of *Fit2* in mouse skeletal muscle reportedly leads to increased energy expenditure, indicating an unexpected function of FIT2 in regulatory aspects of energy metabolism (Miranda et al*.*, 2011), which might be crucial in tissues that are affected in the described individuals. In connection to this, it is tempting to speculate that altered (regulation of) mitochondrial function is part of the molecular mechanisms of the disease as indications are increasing for a functional connection between LDs, and thus FITM2, and other organelles including mitochondria (Barbosa et al*.,* 2015). Interestingly, mitochondrial dysfunction is indicated to underlie some other deafness-dystonia syndromes e.g. Mohr–Tranebjaerg syndrome with defects in *TIMM8A*. TIMM8A is located in the mitochondrial intermembrane space and functions in mitochondrial morphology (Engl et al*.,* 2012). Alternative and/or additional pathogenic mechanisms for the presented syndrome might be related to ER-stress, analogous to the disease mechanism of motor neuropathies that arise from gain-of-function mutations in *BSCL2*, which encodes seipin, a protein that functions in LD biogenesis (Ito and Suzuki, 2009; Cartwright et al*.,* 2015). Additionally, recently proposed roles of LDs in, e.g. immunity, modulation of nuclear functions, protein degradation, autophagy and lipid signaling might contribute to the pathogenesis of the syndrome (Welte, 2015; Pol et al*.*, 2014). Further studies will be needed to elucidate the molecular mechanisms underlying the syndrome.

In conclusion, we have described a novel deafness-dystonia syndrome that is causally related to a loss-of-function mutation in *FITM2*, the phenotypic effects of which are recapitulated in a *Drosophila* model. The phenotype of the affected individuals suggests that in humans, FITM2 function extends beyond its roles in neutral lipid storage and metabolism.

## MATERIALS AND METHODS

### Patient evaluation

Written informed consent was obtained from individuals I:1 and I:2 and included consent for themselves and for their offspring who were not able to sign and/or were younger than 18 years when the genetic studies were performed. The human subjects review boards of the Institute of Biomedical and Genetic Engineering, Islamabad, Pakistan, the medical ethics committee of the Radboud University Medical Center, Nijmegen, the Netherlands (2010-418), and the Domain Specific Review Board for ethics of the National Healthcare Group, Singapore (2012/00295) approved the study protocol. All clinical investigations were performed according to the principles expressed in the Declaration of Helsinki.

Patients of the presented family (W09-1008; Fig. 1A) were evaluated by medical specialists in pediatrics, otorhinolaryngology, and neurology.

Tympanometry, pure-tone audiometry, brainstem-evoked response audiometry (BERA), magnetic resonance imaging (MRI) of the brain, measurements of fasting levels of glucose and triglycerides and of other molecules in serum and neurophysiological evaluations were performed according to standard protocols. Muscle tissue derived from a musculus vastus lateralis biopsy was embedded in paraffin and stained with hematoxylin-eosin according to standard protocols.

#### MRI of abdomen

The abdominal MR images were acquired from a 3T MR scanner (Tim Trio Siemens) using two-point Dixon sequence repetition time (TR)=5.28 ms, echo time (TE)1=2.45 ms, TE2=3.68 ms, FA=9°, bandwidth1=500 Hz Px^−1^, bandwidth2=780 Hz Px^−1^ and Siemens body matrix coil after anatomical localization. For the parent (I:1), 80 axial slices with 3 mm thickness, 0.6 mm interslice gap and in-plane resolution of 1.25×1.25 mm were acquired and 52 slices with in-plane resolution of 1.02×1.02 mm were acquired for two affected children (II:5 and II:6). A fully automated segmentation technique was employed to segment and quantify the abdominal fat volumes between the first (L1) and fifth (L5) lumbar vertebrae (Sadananthan et al*.*, 2015). First, the fat tissues were separated from non-fat tissues by intensity thresholding. The extracted fat tissues were then classified into subcutaneous (SAT) and visceral (VAT) adipose tissues using graph theoretic segmentation.

#### MR spectroscopy of the liver

Fat content in the liver was determined using ^1^H magnetic resonance spectroscopy (MRS). The liver spectra were obtained from a 2×2×2 cm^3^ voxel from two locations (right and left lobes) using a point-resolved spectroscopy (PRESS) sequence (TE=30 ms, TR=2000 ms) and a Siemens body matrix coil. The acquired spectra were fitted using the linear combination of model spectra (LCModel) (Sadananthan et al*.*, 2015; Provencher, 1993). The liver fat was determined from the concentration of methyl and methylene groups of lipids and the unsuppressed water signal and corrected for T2 losses (Cowin et al*.*, 2008).

### Genetic analyses

#### SNP genotyping

Genomic DNA was isolated from peripheral blood lymphocytes by standard procedures. All family members were genotyped employing the HumanOmniExpress BeadChip v1.1 (Illumina) arrays with 719,659 SNPs. Homozygosity mapping using 696,513 autosomal SNPs with genotype calls in all samples (364,151 polymorphic) using PLINK v1.07 was performed (Purcell et al*.*, 2007). Overlapping homozygous regions >5 Mb in size present in all affected and absent in the unaffected individuals were selected. Family relationships among genotyped individuals using identity-by-descent checks were performed. We further conducted a genome-wide linkage scan using MERLIN 1.1.2 on a pruned subset of 53,028 independent SNPs (defined as pair-wise r^2^<0.1), as the inclusion of SNPs in strong linkage disequilibrium is known to result in inflation of linkage tests, based on inheritance of the same ancestral mutant allele (0.001) from both parents (coded as first cousins) under a recessive model (Abecasis et al*.*, 2002).

We confirmed the reported familial relationships among genotyped samples using PLINK identity by descent (IBD) analysis (--genome), with parent-offspring pairs sharing ∼50% of alleles IBD and ∼100% of loci sharing 1 out of 2 alleles IBD, and full sibling pairs sharing ∼50% alleles IBD with the expected ∼25% of loci sharing 0 alleles IBD, ∼50% sharing 1 allele IBD and ∼25% sharing 2 alleles IBD.

#### Sequence analysis; WES and Sanger sequencing

Whole-exome sequencing was performed in the non-affected parents, I:1 and I:2, and in two affected siblings, II:5 and II:6, using the Nimblegen SeqCap EZ exome v3 kit and protocol (Roche). The captured libraries were barcoded, pooled and sequenced on a single lane in a multiplexed 2×101 bp Illumina HiSeq 2000 sequencing run. Reads were mapped against the UCSC Genome Browser Hg19 assembly (build 37) using BWA v1.7 and variants were called using the Genome Analysis Toolkit (GATK) v2 (Broad Institute) following the recommended guidelines. Mean sequence depth was 79.5× with >96% of the exome covered by ≥10 reads. Identified variants were evaluated with the SIFT tool (http://sift.jcvi.org/) and checked against public databases of exomic or genomic variants [1000 genomes, HapMap and NHLBI exome variant server (http://evs.gs.washington.edu/EVS/)].

Primers for amplification of exons and exon-intron boundaries of *FITM2* (uc002xlr.1) were designed with ExonPrimer (Helmholz Center Munich, Institute of Human Genetics; https://ihg.helmholtz-muenchen.de/ihg/ExonPrimer.html). Amplification by PCR was performed on 40 ng of genomic DNA with Taq DNA polymerase (Roche) or Amplitaq (Life Technologies). Primer sequences are provided in Table S1. PCR fragments were purified with NucleoFast 96 PCR plates (Clontech) in accordance with the manufacturer's protocol. Sequence analysis was performed with the ABI PRISM BigDye Terminator Cycle Sequencing V2.0 Ready Reaction kit and analyzed with the ABI PRISM 3730 DNA analyzer (Applied Biosystems). Presence of the *FITM2* c.4G>T transversion was determined in 137 ethnically matched healthy controls by restriction analysis of amplicons encompassing *FITM2* exon 1 (primers as indicated in Table S1), which were purified as described above and digested with *Nsp*I (New England Biolabs) in accordance with the manufacturer's protocol. Restriction fragments were analyzed on 2% agarose gels. The mutation removes a restriction site. The absence of the *FITM2* c.4G>T variant was also verified in the Nijmegen WES database (5031 exomes) and ExAC (65,000 exomes). The variant was submitted to the Leiden open variation database (LOVD; ID #0000079006; http://databases.lovd.nl/).

### Expression analysis of FITM2 in HEK293T cells

Wild-type and c.4G>T *FITM2* cDNA were cloned in an expression vector containing a C-terminal Streptavidin-FLAG-tag (SF-TAP) (Gloeckner et al*.*, 2007) using Gateway Cloning technology (Life Technologies) according to the manufacturer's instructions. Only the protein-coding *FITM2* sequences are represented in the construct (NM_001080472.1). HEK293T cells were cultured in high-glucose DMEM AQmedia (Sigma Aldrich), supplemented with 10% FCS, 1% penicillin/streptomycin and 1 mM sodium pyruvate. For DNA transfections, HEK293T cells were seeded in 6-well plates, grown overnight, and transfected with 2 µg of plasmid using PEI transfection reagent (Merck Millipore). Twenty-four hours after transfection cells were washed with PBS and lysed on ice in lysis buffer [50 mM Tris-HCl pH 7.5, 150 mM NaCl, 0.5% Triton X-100 supplemented with complete protease inhibitor cocktail (Roche)]. Affinity purification of SF-TAP-tagged proteins was performed on cleared lysates using anti-FLAG M2 affinity gel (Sigma Aldrich). Lysates were incubated for four hours at 4°C and subsequently precipitated by centrifugation and washed three times in lysis buffer. Protein lysates and affinity purified samples were analyzed on western blots of NuPAGE Novex 12% Bis-Tris Protein Gels (Life Technologies) and imaged using the Odyssey Infrared Imaging System (LI-COR). Tagged molecules were detected by anti-FLAG antibodies (1:1000; Sigma Aldrich, F7425) and IRDye800 goat-anti-rabbit IgG (1:10,000; LI-COR, 926-32211) as described (Roosing et al*.*, 2014).

### RNAi and phenotypic analyses in *Drosophila melanogaster*

#### *Drosophila melanogaster* stocks and maintenance

We modeled loss of human *FITM2* by constitutive knockdown in *Drosophila*, exploiting two independent, inducible RNAi constructs against both isoforms encoded by *CG10671*, *CG10671-RA* and *CG10671-RB*, and the *UAS*-GAL4 system (Brand and Perrimon, 1993; Dietzl et al*.*, 2007). Experiments were replicated in multiple stocks, two from the GD RNAi library (v44433 and v44435 harboring the RNAi construct GD3580; referred to as *Fitm* RNAi-1A and *Fitm* RNAi-1B, respectively) with the corresponding genetic background control (v60000; Control-1) and one from the KK RNAi library (v109895 with the RNAi construct KK107999; *Fitm* RNAi-2) with the corresponding genetic background control (v60100; Control-2). The stocks were obtained from the Vienna *Drosophila* RNAi Centre (VDRC; http://stockcenter.vdrc.at/control/main) (Dietzl et al*.*, 2007).

RNAi expression was induced by a variety of GAL4 driver lines, which carry a tissue-specific promoter driving the expression of GAL4. The *w; C7-*GAL4*; UAS-Dcr-2* (fat body expression) line was kindly provided by Marek Jindra (Rynes et al*.*, 2012). The GAL4 promoter driver lines, *w;; αTub84B-*GAL4*/ TM6C, Sb^1^ Tb^1^* (5138) (ubiquitous expression) and *w,* *UAS-Dcr-2; Mef2-*GAL4 (25756) (preferentially expressed in skeletal muscle) were obtained from the Bloomington *Drosophila* Stock Center (BDSC; http://flystocks.bio.indiana.edu/). The *w;; elav-*GAL4 (8760) line was obtained from the BDSC and combined with *w; UAS*-*Dcr-2* (60009) from VDRC to create *w; UAS*-*Dcr-2*; *elav-*GAL4 (pan-neuronal expression). A copy of *UAS*-*Dcr-2* was included to improve the efficiency of knockdown (Dietzl et al*.*, 2007). The *w, UAS-Dcr-2; 477-*GAL4*,* *UAS-*mCD8::GFP*;* *ppk-*GAL4 driver [expression in class IV dendritic arborization (da) neurons] was assembled from *yw, 477*-GAL4*;* *UAS*-mCD8::GFP (8768) and *w;; ppk*-GAL4 (32079), both from BDSC. Crosses were maintained according to standard procedures at 28°C.

#### Confirmation of *Fitm* knockdown by qRT-PCR

In order to evaluate the efficiency of RNAi-induced knockdown, *Fitm* RNAi-1A, *Fitm* RNAi-1B and *Fitm* RNAi-2 lines were crossed to the *αTub84B*-GAL4 driver (ubiquitous). One-day-old males of the appropriate genotype were selected for qRT-PCR evaluation of *Fitm* RNAi-1A and RNAi-2 lines, one-day-old females for evaluation of knockdown using the X-linked *Fitm* RNAi-1B line. Extraction of mRNA, cDNA synthesis and qPCR were performed as previously described (Mukhopadhyay et al*.*, 2010). The gene encoding RNA polymerase II (*RpII215*) was used as a reference gene. Primer pairs for amplification of both *Fitm* isoforms (*CG10671-RA* and *CG10671-RB*) and *RpII215* transcripts were designed using ExonPrimer software. For each genotype, three biological and two technical replicates were performed. Differential gene expression was calculated using the 2^ΔΔCt^ method (Livak and Schmittgen, 2001). One-way ANOVA (GraphPad Prism version 5.00 for Windows) was employed for calculations of *P*-values.

#### Negative geotaxis assay

*Fitm* RNAi lines and the corresponding genetic background control lines were crossed to the *αTub84B*-GAL4, *Mef2*-GAL4 and the *C7*-GAL4 driver lines. Female and male progeny of the appropriate genotypes and age were subjected to the negative geotaxis assay (Benzer, 1967; Ali et al*.*, 2011). Locomotor climbing abilities where observed after tapping down the flies in the vials. The natural response of flies is to climb up the vials after tapping. In case of locomotor impairment, flies exhibit slower climbing behavior or non-ability to climb.

All behavioral tests were performed at room temperature under standard light conditions. Aged flies were transferred to fresh food vials every three to four days.

#### Island assay

*Fitm* RNAi lines and the corresponding genetic background control lines were crossed to the *αTub84B*-GAL4 (ubiquitous), the *Mef2*-GAL4 (preferentially expressed in skeletal muscle) and the *C7*-GAL4 (fat body) driver lines. Female and male progeny of the appropriate genotypes and age were subjected to the island assay (Schmidt et al*.*, 2012). In brief, flies were simultaneously released onto a platform in the middle of a soap bath and their escape response was videotaped. Flies remaining on the platform after 10 s were manually counted. Flight ability, wing and leg movements were visually evaluated (Lee et al*.*, 2009). If an abnormal locomotion behavior was found, at least one additional experiment was performed to confirm the observed behavioral defects. The SPSS statistics 20 package (IBM) was used for the ANOVA statistical comparisons.

All behavioral experiments were performed at room temperature under standard light conditions. Aged flies were transferred to fresh food vials every three to four days.

#### Dendritic morphology of class IV dendritic arborization neurons

Male third instar larvae were dissected following a ventral midline incision for imaging of the dorsal class IV ddaC neurons. The *Fitm* RNAi-1A and *Fitm* RNAi-2 lines and the corresponding controls were crossed to *w, UAS-Dcr-2; 477-*GAL4*,* *UAS-*mCD8::GFP*; ppk-*GAL4 driver line. Dendritic neurons were stained with rat anti-mouse CD8a (1:100; Thermo Fisher Scientific, MCD0800) and goat anti-rat Alexa Fluor 488 (1:200; Thermo Fisher Scientific, A-11006). Z-stack images were taken at a Zeiss LSM 510 confocal microscope with a 20× objective. Z-stacks were imported into NeuronStudio (version 0.9.92; Wearne et al*.*, 2005; http://research.mssm.edu/cnic/tools-ns.html) for generation of neuronal reconstructions and Sholl analysis (10 μm interval). Tracing files were analyzed with L-Measure (version 5.2; Scorcioni et al*.*, 2008) and statistical significance was analyzed using the one-sample *t*-test in GraphPad Prism. Data was collected from larvae selected from two independent experiments.

#### Hearing test

To assess sound responses of the *Drosophila* Johnston's organ (JO), antennal vibrations and ensuing antennal nerve potentials were measured in adult flies three days after eclosion as previously described (Göpfert et al*.*, 2006). The *Fitm* RNAi-1A, *Fitm* RNAi-1B and *Fitm* RNAi-2 lines and the corresponding controls were crossed to the *elav*-GAL4 and *αTub84B*-GAL4 drivers. In the *Fitm* RNAi-1B line, the RNAi construct was located in the X chromosome and therefore only female progeny were evaluated of this condition and its control. When using the lines *Fitm* RNAi-1A, Control-1, *Fitm* RNAi-2 and Control-2, both females and males were selected. In brief, antennal vibrations were monitored at the tip of the antennal arista using a PSV-400 scanning laser Doppler vibrometer (LDV) with an OFV-500 close-up unit (Polytec GmbH). Pure tones adjusted to the mechanical best frequency of the antenna were used as sound stimuli. The resulting sound particle velocity was measured with an Emkay NR3158 pressure gradient microphone (distributed by Knowles Electronics Inc.) at the position of the fly. In line with previous reports (Göpfert et al*.*, 2006), the individual best frequency of each antenna was determined from the power spectrum of its mechanical free fluctuations in the absence of sound stimulation, and tone-evoked antennal vibration amplitudes were measured as Fourier amplitudes at the frequency of sound stimulation. Ensuing nerve potentials were measured in the form of compound action potentials (CAPs) from the axonal projections of JO neurons in the antennal nerve via an electrolytically tapered tungsten electrode inserted between the antenna and the head (Effertz et al*.,* 2011; Senthilan et al*.*, 2012). A tungsten wire inserted into the thorax served as indifferent electrode. CAP amplitudes were plotted against the corresponding sound particle velocities. Hill fits were used to determine the sound particle velocity threshold of the CAPs, whereby the particle velocity corresponding to 10% of the maximum amplitude approached by the fit was used as the threshold criterion. To quantify the amplification gain exerted by motile responses of JO neurons, the antenna's mechanical sensitivity, measured as antennal displacement amplitudes normalized to the corresponding sound particle velocities, was plotted against the particle velocity of the stimulus tones. The amplification gain was then measured as the ratio between the antenna's mechanical sensitivity in the low and high intensity regimes (Göpfert et al*.*, 2006; Senthilan et al*.*, 2012). Data analysis was performed using Polytec-VIB (Polytec GmbH), Spike 2 (Cambridge Electronic Design), Excel 2007 (Microsoft), SigmaPlot 10 (Systat Software) and Prism (GraphPad).

#### Fat body analysis

*Fitm* RNAi lines and the corresponding controls were crossed to the *C7*-GAL4 driver line (fat body). Progeny was transferred to vials with fresh food every two days. Fat bodies of female flies were dissected at the indicated adult age, fixed in PBS with 3.7% paraformaldehyde for 20 min, rinsed with PBS, stained with Bodipy (1:2000; Life Technologies, C3922) for 20 min at room temperature, and mounted in Vectashield with DAPI (Vector Laboratories). Pictures were obtained using a Zeiss Axio Imager ZI fluorescence microscope (Zeiss). Data was collected from flies selected from two independent experiments. LD area was assessed using Fiji (NIH). A minimum of two random regions of interest (ROI) of 35.5 µm^2^ were created from each image and the area of the LDs contained in the ROI was retrieved and analyzed. Statistical significance was calculated by using ANOVA with the Tukey correction for multiple testing incorporated in GraphPad Prism.
